# The impact of weight change on suicide mortality: a nationwide population-based cohort study of 2 million Koreans

**DOI:** 10.1186/s13098-024-01559-7

**Published:** 2025-01-19

**Authors:** Kyuho Kim, Jin-Hyung Jung, Yoo Hyun Um, Yu-Bae Ahn, Seung-Hyun Ko, Kyungdo Han, Jae-Seung Yun

**Affiliations:** 1 Division of Endocrinology and Metabolism, Department of Internal Medicine, St. Vincent’s Hospital, College of Medicine, The Catholic University of Korea, 222 Banpo-daero, Seocho-gu, Seoul, 06591 Republic of Korea; 2 Samsung Biomedical Research Institute, Sungkyunkwan University School of Medicine, Suwon, Republic of Korea; 3 Department of Psychiatry, St. Vincent’s Hospital, College of Medicine, The Catholic University of Korea, Seoul, Republic of Korea; 4 Department of Statistics and Actuarial Science, Soongsil University, 369 Sangdo-ro, Dongjak-gu, Seoul, 06987 Republic of Korea

**Keywords:** Mortality, Suicide, Weight change, Weight gain, Weight loss

## Abstract

**Background:**

Previous studies have shown that weight change has a reverse J-shape association with all-cause mortality. However, its association with suicide mortality remains undetermined. In this study, we investigated the association between weight change and suicide mortality using a large-scale, population-based cohort from the Korean National Health Insurance Service database.

**Methods:**

A total of 2,103,525 subjects aged ≥ 20 years who underwent a general health screening program twice in the 2-year interval between 2007 and 2009 were included. Subjects were categorized into five groups according to the percent weight change during this period: severe weight loss (< − 15.0%), moderate weight loss (− 15.0 to < − 5.0%), weight stable (− 5.0 to < 5.0%), moderate weight gain (5.0 to < 15.0%), and severe weight gain (≥ 15.0%).

**Results:**

During a median follow-up of 11.3 years, 6,179 cases (0.3%) of suicide mortality occurred. Weight change was associated with increased suicide mortality in a reverse J-shaped curve, even after adjustment for covariates. In particular, those with severe weight loss or gain showed 1.8-fold or 1.6-fold increased risk of suicide mortality, respectively. This reverse J-shaped association was consistently observed in subgroup analyses considering age, sex, depression, cancer, and BMI category.

**Conclusions:**

Moderate to severe weight change within a 2-year interval is associated with increased risk of suicide mortality. To better understand the mechanisms through which weight change affects suicide mortality, studies incorporating information on weight change intentions, medications, weight change-related medical conditions are needed.

**Supplementary Information:**

The online version contains supplementary material available at 10.1186/s13098-024-01559-7.

## Introduction

Overweight or obesity is a risk factor for cardiovascular disease (CVD) and metabolic diseases such as type 2 diabetes, hypertension, and dyslipidemia [1, 2]. Body mass index (BMI) is a popular indicator of an individuals’ fatness [3], and several studies have consistently shown that both high BMI (overweight or obesity) and low BMI (underweight) are associated with increased mortality, following J-shaped or U-shaped curves [4–6].

Whether weight loss or weight gain can influence mortality is an intriguing question; several studies have indeed demonstrated that both weight loss [7, 8] and weight gain [9, 10] are associated with increased mortality. In addition, a nationwide population-based study including approximately 10 million Korean adults demonstrated that weight loss or gain > 5.0% within a 4-year interval is associated with increased risk of all-cause mortality [11]. However, the association between weight change and cause-specific mortality has not been studied well.

According to the World Health Organization (WHO), suicide accounted for 1.3% of all deaths in 2019 [12]. In United States, it was the 10th leading cause of death overall, the 2nd in people aged 10–34 years, and the 5th in people aged 35–54 years [13]. In South Korea, it is the 5th leading cause of death overall, the 1st in people aged 10–39, and 2nd in people aged 40–59 years [14]. A previous prospective cohort study including 18,784 men aged 40–69 years in the United Kingdom showed unexplained weight loss to be associated with an increased risk of suicide mortality [15]. However, the study was limited in that only men were included, and information was lacking about the amount of weight loss and diagnoses of psychiatric disorders such as depression. In addition, research is scarce concerning how much weight change increases suicide mortality among subjects of differing BMI categories. Considering that the European Medicines Agency has started to review data on the risk of suicidal thoughts with anti-obesity medications such as semaglutide and liraglutide [16], there is a need to examine whether weight change is associated with suicide mortality according to BMI category.

Therefore, we aimed to investigate the association between weight change and suicide mortality using a large-scale, population-based cohort derived from the Korean National Health Insurance Service (NHIS) general health screening program. In addition, we performed subgroup analyses according to age, sex, diabetes, depression, cancer, and BMI category.

## Methods

### Study population

The NHIS is a mandatory national health insurance managed by the Korean government, providing health insurance to approximately 97.0% of the Korean population. The NHIS database includes data on demographics, diagnoses, prescriptions, medical procedures, inpatient and outpatient service records, and death records. The NHIS offers a general health screening program biannually for all insured Koreans, with the exception of non-office workers who receive the general health screening program annually [17]. The proportion of participants in the general health screening program relative to the eligible population has been approximately 75% over the past ten years [18]. The general health screening program includes tests for cardiovascular and cerebrovascular diseases, including hypertension, diabetes mellitus, dyslipidemia, and obesity, and for other diseases such as liver disease, psychiatric disorders, and geriatric diseases [19].

**Fig. 1:**
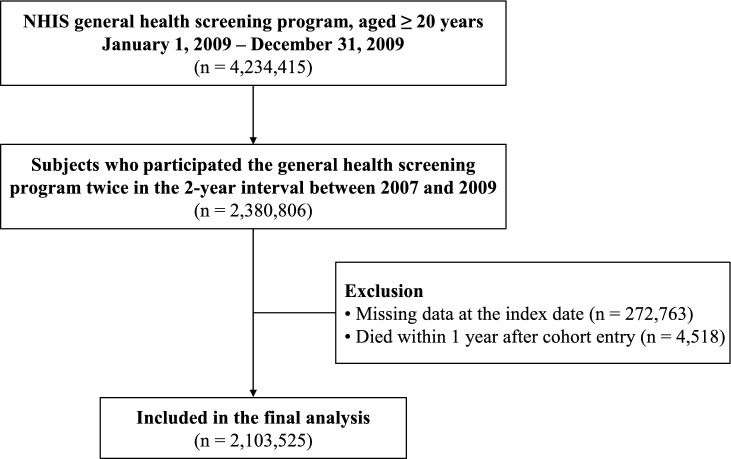
A flowchart of the study population enrollment. NHIS denotes National Health Insurance Service

We identified 4,234,415 subjects aged ≥ 20 years who underwent the general health screening program through the NHIS from January 1, 2009 to December 31, 2009 (index date). Among these, we included subjects who had also participated in the general health screening program 2 years before (in 2007) (N = 2,380,806). We excluded subjects with missing data for one or more variables in the general health screening program at the index date (N = 272,763). In addition, we excluded subjects who died within 1 year after cohort entry (N = 4,518). Ultimately, a total of 2,103,525 subjects were included and followed until the occurrence of suicide death, or December 31, 2021 (Fig. 1).

This study was approved by the Institutional Review Board of The Catholic University of Korea, St. Vincent’s Hospital (VC23ZISE0218), and informed consent was waived. The study was conducted in accordance with the principles of the Declaration of Helsinki.

### Definition of weight change

Weight change was calculated as percent change between 2007 and 2009: (100 × [weight at 2009—weight at 2007]/weight at 2007). We categorized subjects into five groups based on weight change, with reference to previous studies [11, 20, 21]: severe weight loss (weight change < − 15.0%), moderate weight loss (weight change − 15.0 to < − 5.0%), weight stable (weight change − 5.0 to < 5.0%), moderate weight gain (weight change 5.0 to < 15.0%), and severe weight gain (weight change ≥ 15.0%).

### Definition of variables and end point

Demographic data, anthropometric measurements, laboratory results, and comorbidities were obtained during the general health screening program in 2009 (index date). Healthcare professionals measured anthropometric indices (BMI, waist circumference, and blood pressure [BP]) during the general health screening program. Blood pressure was measured using a standard sphygmomanometer after at least 5 min of rest. Blood samples were collected after overnight fasting, and serum glucose and total cholesterol were measured. Information about smoking status, alcohol consumption, and exercise were obtained using self-reported questionnaires. Smoking status was classified as nonsmoker, ex-smoker, or current smoker. Alcohol drinker was defined when subjects had alcohol intake > 0 g per day. Regular exercise was defined as high-intensity activities ≥ 3 times a week or moderate intensity activities ≥ 5 times a week. Income was classified into quartiles (Q): Q1 (low income), Q2, Q3, and Q4. Obesity was defined as a BMI of ≥ 25 kg/m^2^ according to the guidelines of the Korean Society for the Study of Obesity [22]. Diabetes was defined as at least one claim per year for the prescription of antidiabetic medications under the International Statistical Classification of Diseases, 10th revision (ICD-10) codes E11–E14, or fasting glucose level ≥ 126 mg/dL at the time of health screening. Hypertension was defined as systolic BP ≥ 140 mmHg, diastolic BP ≥ 90 mmHg, the ICD-10 codes I10-13 or I15, or prescription of antihypertensive medications. Dyslipidemia was defined as a total cholesterol level ≥ 240 mg/dL, the ICD-10 code E78, or prescription of lipid-lowering medications. Chronic kidney disease (CKD) was defined based on the estimated glomerular filtration rate (eGFR) calculated by the Modification of Diet in Renal Disease formula, with eGFR < 60 mL/min/1.73 m^2^ considered CKD. Depression was defined as the ICD-10 code F32 or F33, as previously described [23–25]. Eating disorder was defined as the ICD-10 code F50. Substance use disorder was defined as the ICD-10 code F10-F19. The NHIS established a special co-payment service to enhance health coverage and relieve the financial burden of patients with cancer. Because enrollment in this co-payment service is indicated by a special reimbursement code for cancer (V193) and requires a medical certificate from a physician, the cancer diagnoses are considered to be sufficiently reliable. Therefore, cancer was defined using the ICD-10 ‘C’ code and the cancer reimbursement code (V193), as previously described [26, 27].

The endpoint was suicide mortality during the follow-up period. Suicide deaths were identified by ICD-10 codes X60–X84, as recorded in the government mortality database (Statistics Korea), as previously described [28]. Follow-up duration was defined as the interval from the index date to the date of suicide mortality, or until December 2021, whichever came first.

### Statistical analysis

Data were expressed as the mean ± standard deviation, median (interquartile range), or number with percentage (%). Baseline characteristics were compared between groups using ANOVA for continuous variables and the chi-square test for categorical variables. The incidence rate (IR) of suicide mortality was calculated by dividing the number of events per 1,000 person-years. Hazard ratios (HRs) and 95% confidence intervals (CIs) for suicide mortality according to the 5 weight change categories were calculated using multivariable Cox proportional hazard regression models.

Model 1 was non-adjusted. Model 2 was adjusted for age, sex, income (Q1), smoking, alcohol drinking, and regular exercise. Model 3 was adjusted for model 2 plus diabetes, hypertension, dyslipidemia, CKD, and depression. Model 4 was adjusted for model 3 plus weight at the 2009 screening [29]. Model 5 was adjusted for model 4 plus eating disorder and substance use disorder. Subgroup analyses were conducted based on age, sex, diabetes, depression, and cancer, after adjusting for age, sex, income (Q1), smoking, alcohol consumption, regular exercise, diabetes, hypertension, dyslipidemia, chronic kidney disease, depression, and weight at the 2009 screening. Given that waist circumference was positively associated with mortality across all BMI categories examined [30], waist circumference was adjusted instead of weight at the 2009 screening in the BMI subgroup analysis.

All analyses were performed using SAS software version 9.3 (SAS Institute, Cary, NC, USA). A two-sided P < 0.05 was considered statistically significant without adjustment for multiple comparisons.

## Results

### Baseline characteristics

Subjects were 48.0 ± 13.6 years old, with body weight of 64.4 ± 11.5 kg and BMI of 23.8 ± 3.1 kg/m^2^. Obesity was present in 695,159 (33.1%) subjects, diabetes in 180,616 (8.6%), hypertension in 545,535 (25.9%), dyslipidemia in 375,378 (17.9%), CKD in 156,910 (7.5%), and depression in 62,978 (3.0%). Subjects in the weight loss groups were older, had higher fasting glucose levels, lower total cholesterol levels, and were less likely to be smokers or alcohol drinkers. Additionally, they had a higher prevalence of diabetes, hypertension, CKD, and cancer compared to those in the weight stable group. Subjects in the weight gain groups were younger, had lower fasting glucose levels, higher total cholesterol levels, and were less likely to be regular exercisers. Additionally, they had a lower prevalence of diabetes and hypertension compared to those in the weight stable group. Compared to those in the weight stable group, subjects in the weight gain or weight loss groups were less likely to be male and had a higher prevalence of low income (Q1) status and depression (Table 1).

**Table 1:** Baseline characteristics of the study population in 2009

	Total	Severe weight loss (weight change < − 15.0%)	Moderate weight loss (weight change − 15.0 to < − 5.0%)	Weight stable (weight change − 5.0 to < 5.0%)	Moderate weight gain (weight change 5.0% to < 15.0%)	Severe weight gain (weight change ≥ 15.0%)	P for trend
	(N = 2,103,525)	(N = 10,713)	(N = 219,740)	(N = 1,565,679)	(N = 288,375)	(N = 19,018)	
Age (years)	48.0 ± 13.6	51.6 ± 17.5	50.1 ± 14.9	48.3 ± 13.2	44.7 ± 13.8	43.8 ± 16.2	< 0.001
Age group—no. (%)							< 0.001
20–39	640,919 (30.5)	3,349 (31.3)	60,294 (27.4)	446,106 (28.5)	121,446 (42.1)	9,724 (51.1)	
40–64	1,178,114 (56.0)	4,306 (40.2)	116,316 (52.9)	914,676 (58.4)	136,355 (47.3)	6,461 (34.0)	
≥ 65	284,492 (13.5)	3,058 (28.5)	43,130 (19.6)	204,897 (13.1)	30,574 (10.6)	2,833 (14.9)	
Men—no. (%)	1,243,764 (59.1)	4,632 (43.2)	114,786 (52.2)	946,221 (60.4)	168,786 (58.5)	9,339 (49.1)	< 0.001
Height (cm)	164.3 ± 9.2	160.5 ± 9.7	162.7 ± 9.4	164.5 ± 9.1	165.0 ± 9.4	163.8 ± 9.5	< 0.001
Weight (kg)	64.4 ± 11.5	54.6 ± 10.8	60.0 ± 10.8	64.6 ± 11.3	66.9 ± 12.0	69.7 ± 13.1	< 0.001
BMI (kg/m^2^)	23.8 ± 3.1	21.1 ± 3.1	22.6 ± 3.0	23.8 ± 3.1	24.5 ± 3.2	25.9 ± 3.9	< 0.001
BMI (kg/m^2^) in 2007	23.7 ± 3.1	26.2 ± 4.1	24.4 ± 3.2	23.8 ± 3.1	22.8 ± 3.0	21.4 ± 3.0	< 0.001
Waist circumference (cm)	80.7 ± 8.9	77.2 ± 9.6	78.2 ± 9.7	80.8 ± 8.8	81.8 ± 8.9	83.6 ± 9.8	< 0.001
Fasting glucose (mg/dL)	97.2 ± 22.8	100.5 ± 37.8	98.7 ± 29.7	97.2 ± 22.1	95.7 ± 19.6	95.7 ± 22.7	< 0.001
Total cholesterol (mg/dL)	195.9 ± 36.4	187.9 ± 37.7	191.6 ± 36.9	196.2 ± 36.2	197.6 ± 36.7	202.1 ± 40.5	< 0.001
Systolic BP (mmHg)	122.7 ± 14.7	121.8 ± 15.9	121.8 ± 15.1	122.8 ± 14.6	122.8 ± 14.6	122.7 ± 15.2	< 0.001
Diastolic BP (mmHg)	76.6 ± 9.9	75.5 ± 10.2	75.8 ± 9.9	76.7 ± 9.8	76.7 ± 9.9	76.6 ± 10.1	< 0.001
Low-income status—no. (%)	329,169 (15.7)	2,033 (19.0)	39,615 (18.0)	239,415 (15.3)	44,879 (15.6)	3,227 (17.0)	< 0.001
Current smoker—no. (%)	551,756 (26.2)	2,109 (19.7)	53,945 (24.6)	412,633 (26.4)	78,628 (27.3)	4,441 (23.4)	< 0.001
Alcohol drinker—no. (%)	1,048,129 (49.8)	3,836 (35.8)	96,321 (43.8)	790,636 (50.5)	149,068 (51.7)	8,268 (43.5)	< 0.001
Regular exercise—no. (%)	402,197 (19.1)	2,109 (19.7)	45,412 (20.7)	305,685 (19.5)	46,396 (16.1)	2,595 (13.6)	< 0.001
Obesity—no. (%)	695,159 (33.1)	1,100 (10.3)	43,238 (19.7)	522,313 (33.4)	118,112 (41.0)	10,396 (54.7)	< 0.001
Diabetes—no. (%)	180,616 (8.6)	1,501 (14.0)	25,312 (11.5)	132,886 (8.5)	19,328 (6.7)	1,589 (8.4)	< 0.001
Hypertension—no. (%)	545,535 (25.9)	3,376 (31.5)	60,850 (27.7)	410,051 (26.2)	66,662 (23.1)	4,596 (24.2)	< 0.001
Dyslipidemia—no. (%)	375,378 (17.9)	1,781 (16.6)	38,240 (17.4)	280,856 (17.9)	50,419 (17.5)	4,082 (21.5)	< 0.001
CKD—no. (%)	156,910 (7.5)	1,078 (10.1)	17,373 (7.9)	116,728 (7.5)	20,240 (7.0)	1,491 (7.8)	< 0.001
Depression—no. (%)	62,978 (3.0)	602 (5.6)	9,473 (4.3)	43,184 (2.8)	8,974 (3.1)	745 (3.9)	< 0.001
Eating disorder—no. (%)	700 (0.0)	7 (0.1)	123 (0.1)	459 (0.0)	98 (0.0)	13 (0.1)	< 0.001
Substance use disorder—no. (%)	4,470 (0.2)	36 (0.3)	667 (0.3)	3,017 (0.2)	680 (0.2)	70 (0.4)	< 0.001
Cancer—no. (%)	26,584 (1.3)	530 (5.0)	4,297 (2.0)	18,367 (1.2)	3,153 (1.1)	237 (1.3)	< 0.001

Data are shown as mean ± standard deviation, median (25–75th percentile), or number (%). BMI denotes body mass index, BP blood pressure, and CKD chronic kidney disease

### Suicide mortality according to weight change

**Fig. 2:**
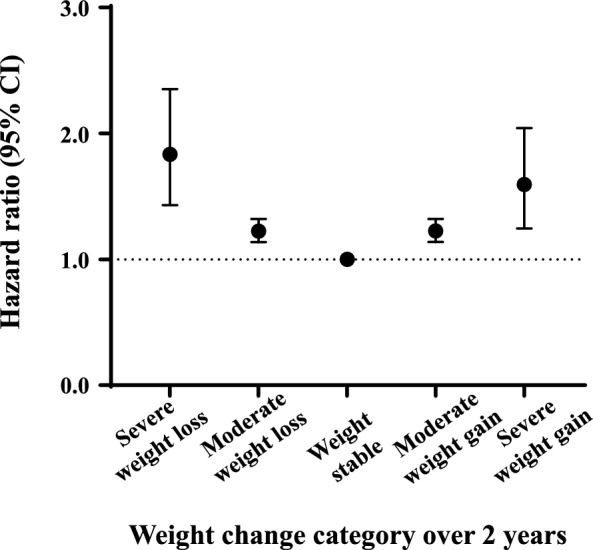
Risk of suicide mortality. Hazard ratios (HRs) with 95% confidence intervals (CIs) are presented as dot and whisker plots after adjusting for covariates: age, sex, income (Q1), smoking, alcohol drinking, regular exercise, diabetes, hypertension, dyslipidemia, chronic kidney disease, depression, weight at the 2009 screening, eating disorder, and substance use disorder. Severe weight loss group (weight change < − 15.0%), moderate weight loss group (weight change − 15.0 to < − 5.0%), weight stable group (weight change − 5.0 to < 5.0%), moderate weight gain (weight change 5.0 to < 15.0%), and severe weight gain (weight change ≥ 15.0%)

Over a median follow-up of 11.3 years (interquartile range, 11.1–11.6), 6,179 cases of suicide mortality occurred. The weight loss group had a higher incidence of suicide mortality than the weight stable or weight gain groups (Additional file 1: Fig. S1). The IR of suicide death was 0.58, 0.35, 0.25, 0.26, and 0.31 per 1,000 person-years for the severe weight loss, moderate weight loss, weight stable, moderate weight gain, and severe weight gain groups, respectively. In the non-adjusted model, severe or moderate weight loss associated with increased risk of suicide mortality. After multivariable adjustment, suicide mortality showed a reverse J-shaped curve according to weight change. Both weight loss (HR, 1.84; 95% CI 1.44–2.36 for the severe weight loss group, and HR, 1.23; 95% CI 1.14–1.32 for the moderate weight loss group) and weight gain (HR, 1.62; 95% CI 1.26–2.07 for the severe weight gain group, and HR, 1.23; 95% CI 1.14–1.33 for the moderate weight gain group) were significantly associated with increased risk of suicide mortality compared with the weight stable group (Table 2 and Fig. 2).

**Table 2:** Association between weight change and suicide mortality

Weight change	Number	Event	IR/1,000 PY	Model 1	Model 2	Model 3	Model 4	Model 5
				HR (95% CI)	HR (95% CI)	HR (95% CI)	HR (95% CI)	HR (95% CI)
< − 15.0%	10,713	64	0.58	2.31 (1.80–2.95)	2.34 (0.83–2.99)	2.25 (1.76–2.88)	1.84 (1.44–2.36)	1.83 (1.43–2.35)
− 15.0 to < − 5.0%	219,740	832	0.35	1.38 (1.29–1.49)	1.36 (1.26–1.46)	1.33 (1.23–1.43)	1.23 (1.14–1.32)	1.22 (1.14–1.32)
− 5.0 to < 5.0%	1,565,679	4,376	0.25	1 (Ref.)	1 (Ref.)	1 (Ref.)	1 (Ref.)	1 (Ref.)
5.0 to < 15.0%	288,375	843	0.26	1.05 (0.97–1.13)	1.21 (1.13–1.30)	1.19 (1.11–1.28)	1.23 (1.14–1.33)	1.23 (1.14–1.32)
≥ 15.0%	19,018	64	0.31	1.23 (0.96–1.57)	1.53 (1.20–1.96)	1.48 (1.16–1.90)	1.62 (1.26–2.07)	1.59 (1.25–2.04)

Model 1: non-adjusted. Model 2: model 1 plus adjustment for age, sex, income (Q1), smoking, alcohol drinking, and regular exercise. Model 3: model 2 plus adjustment for diabetes, hypertension, dyslipidemia, chronic kidney disease, and depression. Model 4: model 3 plus adjustment for weight at the 2009 screening. Model 5: model 4 plus adjustment for eating disorder and substance use disorder

*CI* denotes confidence interval, *HR* hazard ratio, *IR* incidence rate, and *PY* person-years

### Subgroup analyses according to age, sex, diabetes, depression, cancer, and BMI category

The relationship between weight change and risk of suicide mortality did not demonstrate significant interactions with age, sex, depression, cancer, or BMI category, instead showing a consistent trend across these factors. However, a significant interaction between diabetes presence and suicide risk was found (P = 0.03 for interaction), with no significant increase in risk for severe weight loss among patients with diabetes. Conversely, a 1.9-fold increase in the risk of suicide mortality was observed in those with diabetes and severe weight gain (Additional file 1: Table S1 and Figs. S2, S3, S4).

## Discussion

In this study, we investigated the association between body weight changes and suicide mortality. The main findings are summarized as follows. First, approximately 25.6% and 1.4% of this large nation-wide population-based cohort experienced moderate or severe weight changes respectively in the 2-year follow-up period. Second, a reverse J-shaped association between weight change and suicide mortality was observed; especially, those with severe weight loss or weight gain showed 1.8-fold or 1.6-fold increased risk of suicide mortality, respectively. Third, this trend of U-shaped or J-shaped association was consistently observed in subgroup analyses according to sex, age, depression, cancer, and BMI category.

Several previous studies have shown that weight change, whether loss or gain, is associated with increased mortality [11, 31, 32]. However, only a few studies have considered the association between weight change and suicide mortality. Considering that only 24.0% of those who die by suicide had psychiatric diagnoses in the 4-week period prior to death [33], it is helpful to identify those at high risk of suicide based on physiologic indicators along with psychiatric markers. The reverse J-shaped association between weight change and suicide mortality observed in this study, which was independent of depression, suggests that weight change can be used as physiologic indicator for suicide mortality. From another perspective, considering the rise in popularity of semaglutide [34] and bariatric surgery [35] and the recent release of tirzepatide [36], all of which provide for weight loss of ≥ 15.0% in subjects with obesity, it is meaningful to examine the association between weight change and suicide mortality. Due to the large number of subjects and long-term follow-up period, the Korean nationwide database provides a good opportunity to investigate the association between weight change and suicide mortality.

Our observation of increased risk of suicide mortality in association with weight loss is in line with previous findings [15, 37]. A prospective cohort study in the United Kingdom showed unexplained weight loss to be associated with increased risk of suicide mortality [15]. However, that study included only men aged 40–69, and it was limited by not considering depression. Another study in Japan, which analyzed a cohort of 41,746 adults and identified 80 cases of suicide events, revealed weight loss since the age of 20 to be significantly associated with an elevated risk of suicide mortality [38]. Several hypotheses can be suggested as potential reasons for suicide resulting from weight loss. Mental health issues such as appetite suppression due to depression, significant psychological stress, and feelings of lethargy can lead to sudden weight loss, potentially triggering thoughts and behaviors related to suicide [37]. Severe systemic illnesses such as cancer, infectious diseases, and gastrointestinal disorders can also be associated with weight loss. A previous study using data from the National Health and Nutrition Examination Survey demonstrated that subjects losing at least 10% of weight within one year have likely been diagnosed with medical illnesses such as arthritis, diabetes, cancer, cardiovascular disease, liver conditions, and respiratory diseases [39]. Dietary restrictions can also lead to severe weight loss. Our study utilized the NHIS general health screening cohort, which included relatively healthy individuals who underwent consecutive biennial national health examinations. In our findings, rapid weight loss was significantly associated with increased risk of suicide even in individuals without a history of cancer, major metabolic diseases such as diabetes, or depression. In addition, subgroup analyses demonstrated that regardless of baseline BMI category, i.e., normal weight, overweight, or obesity, moderate or severe weight change is associated increased risk of suicide mortality. Conversely, among young adults aged 20–39, those with diabetes, depression, cancer, or obesity class II showed no statistically significant increase in suicide risk associated with severe weight loss. The interpretation of these results may consider factors such as a low occurrence of suicide deaths in these groups, deaths due to factors other than suicide, and potential health improvements resulting from weight loss.

We also found increased risk of suicide mortality to be associated with weight gain in this study. One possible explanation is that weight control failure (weight gain despite weight loss efforts) could increase risk of suicidal ideation [40]. In addition, considering that psychiatric disorders such as schizophrenia [41] and bipolar disorder [42] are risk factors of suicide, and medications for these disorders increases body weight [43, 44], increased suicide mortality associated with weight gain might be attributed to psychiatric disorders. Whereas subjects without diabetes showed a reverse J-shaped association between weight change and suicide mortality, those having diabetes showed a J-shaped association between weight change and suicide mortality; especially, the group with severe weight gain showed 1.9-fold increased risk of suicide mortality. In subjects with type 2 diabetes, weight gain is associated with increased risk of medical illness such as cardiovascular disease [45], and contributes to patient frustration [46], which might increase the risk of suicide mortality.

The current study has several strengths. First, this study is a large-scale population-based cohort study including more than 2 million subjects. This enabled us to perform multiple subgroup analyses with statistical power. Second, the analyses were adjusted for the presence of chronic diseases such as diabetes, hypertension, dyslipidemia, and CKD, and especially the presence of depressive disorder. Nevertheless, this study also has some limitations. First, there is a possibility of selection bias, as the study only included individuals who participated in the general health screening program twice within a two-year period. Those with higher health consciousness are more likely to engage in regular health screenings. Second, because this was an observational study, we could not exclude the possibility of unmeasured confounding factors contributing to the reverse J-shaped association between weight change and suicide mortality. Such factors can include conditions such as cardiovascular disease, heart failure, liver disease, chronic obstructive pulmonary disease, dementia, psychiatric disorders other than depression, as well as marital and employment status. Third, our analyses could not differentiate between intentional and unintentional weight loss. Because intentional weight loss to improve health can lower mortality [47, 48], this might affect the observed association between weight change and suicide mortality. In addition, this study did not include information on weight control behaviors, perceived weight status, suicidal ideation, and suicidal attempt, which were associated with suicide mortality [49, 50]. Fourth, we could not consider the onset time and duration of comorbidities, which might affect weight change. Fifth, because the diagnosis of depressive disorder was identified from claim data, there is a possibility of its underestimation. Sixth, this study did not include information on the use of psychiatric or antidiabetic medications that could have influenced weight changes. Lastly, the study results were derived from only Korean subjects. Therefore, the results cannot be generalized to other ethnicities.

## Conclusions

A reverse J-shaped association was found between weight change and suicide mortality; in particular, severe weight loss or weight gain showed 1.8-fold or 1.6-fold increased risk of suicide mortality, respectively. This association was consistently observed regardless of age, sex, depression, cancer, and BMI category. Our findings suggest that physicians should monitor subjects with severe weight change within a 2-year interval in relation to suicide behavior. Future studies incorporating information on weight change intentions, medications, weight change-related medical conditions are needed to elucidate the underlying mechanisms through which weight change affects suicide mortality.

## Supplementary Information

Additional file 1.

## Data Availability

Data from the Korean National Health Insurance Service (NHIS) cannot be shared publicly due to the violation of patient privacy and the absence of informed consent for data sharing.

## Abbreviations

BP: Blood pressure
BMI: Body mass index
CVD: Cardiovascular disease
CKD: Chronic kidney disease
eGFR: Estimated glomerular filtration rate
HR: Hazard ratio
IR: Incidence rate
ICD-10: International Statistical Classification of Diseases, 10th revision
NHIS: National Health Insurance Service
Q: Quartile
WHO: World Health Organization
