# Supplementary material for: The impact of weight change on suicide mortality: a nationwide population-based cohort study of 2 million Koreans
Source: Diabetol Metab Syndr. 2025 Jan 19;17:20. doi: 10.1186/s13098-024-01559-7 (PMC11743014; doi:10.1186/s13098-024-01559-7)
Supplement: Supplementary file 1 — Additional file 1. [file 13098_2024_1559_MOESM1_ESM.docx]

**Figure S1. Kaplan-Meier curves for the risk of suicide mortality according to weight change.** Kaplan-Meier curves were generated in order to visualize the cumulative incidence probability of suicide mortality.


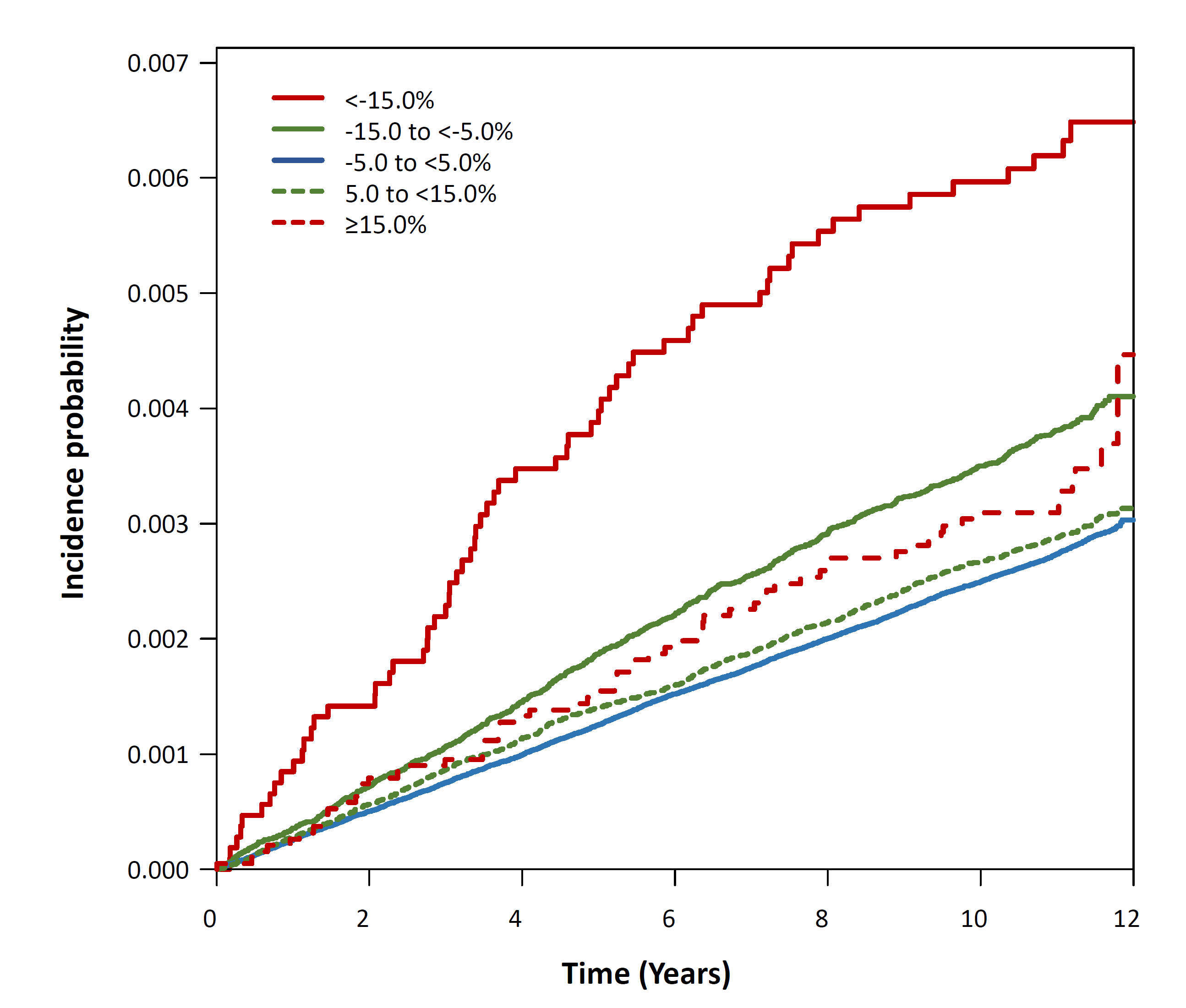


**Figure S2. Subgroup analyses according to age and sex.** Forrest plots of hazard ratios (HRs) and 95% confidence intervals (CIs) adjusted for age, sex, income (Q1), smoking, alcohol drinking, regular exercise, diabetes, hypertension, dyslipidemia, chronic kidney disease, depression, and weight at the 2009 screening.


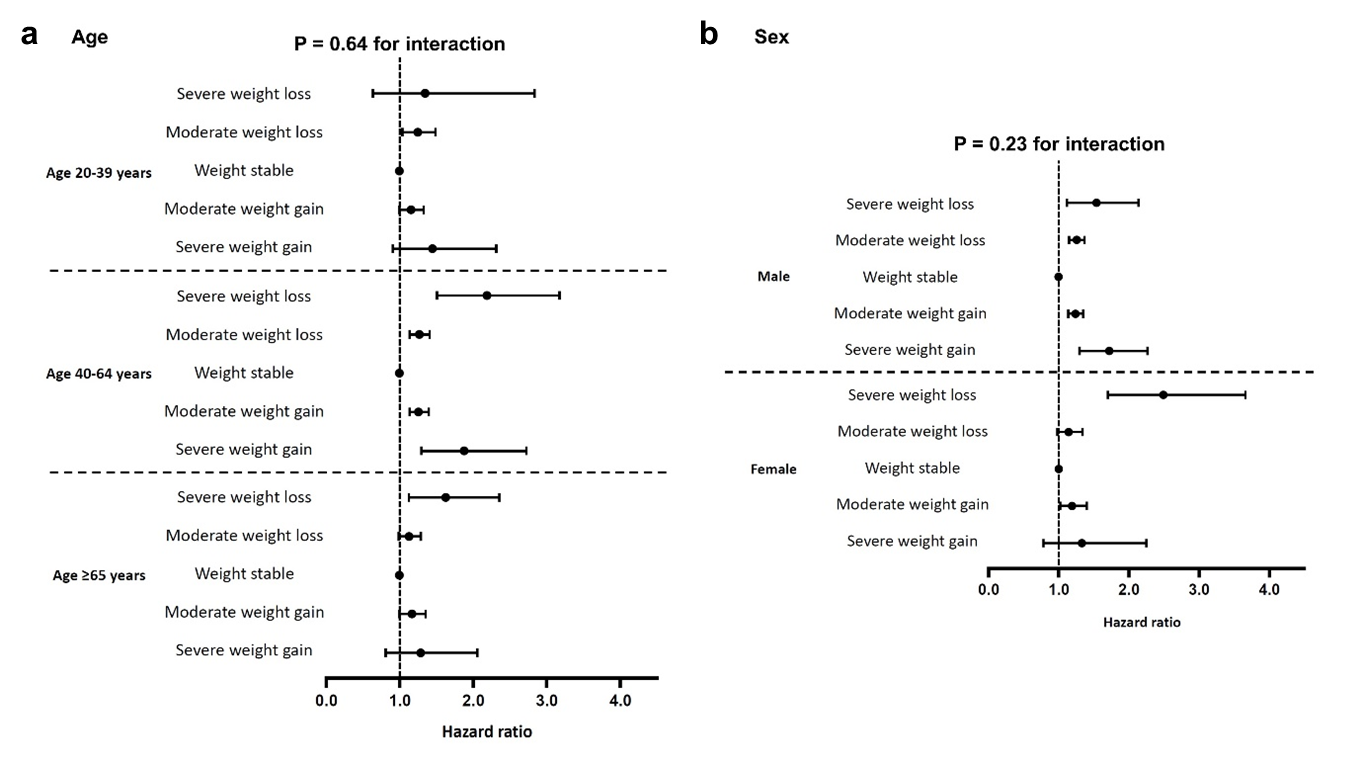


**Figure S3. Subgroup analyses according to diabetes, depression, and cancer.** Forrest plots of hazard ratios (HRs) and 95% confidence intervals (CIs) adjusted for age, sex, income (Q1), smoking, alcohol drinking, regular exercise, diabetes, hypertension, dyslipidemia, chronic kidney disease, depression, and weight at the 2009 screening.

**
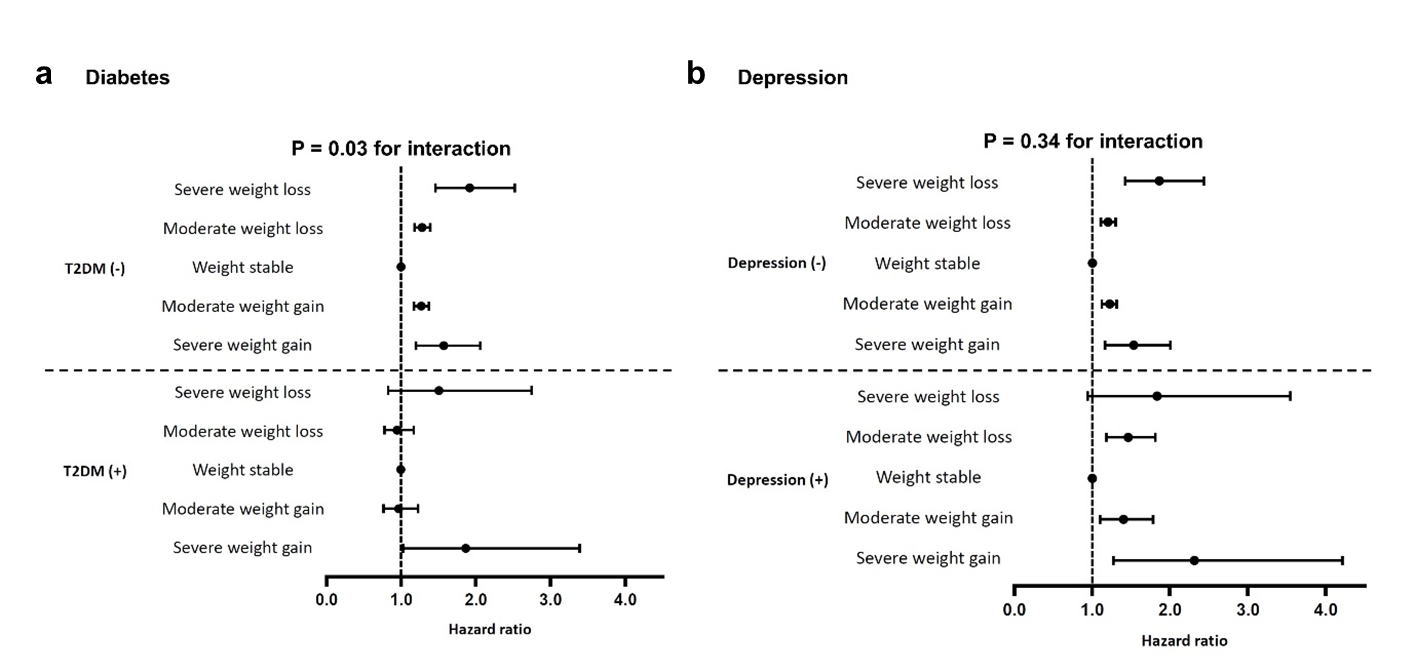
**


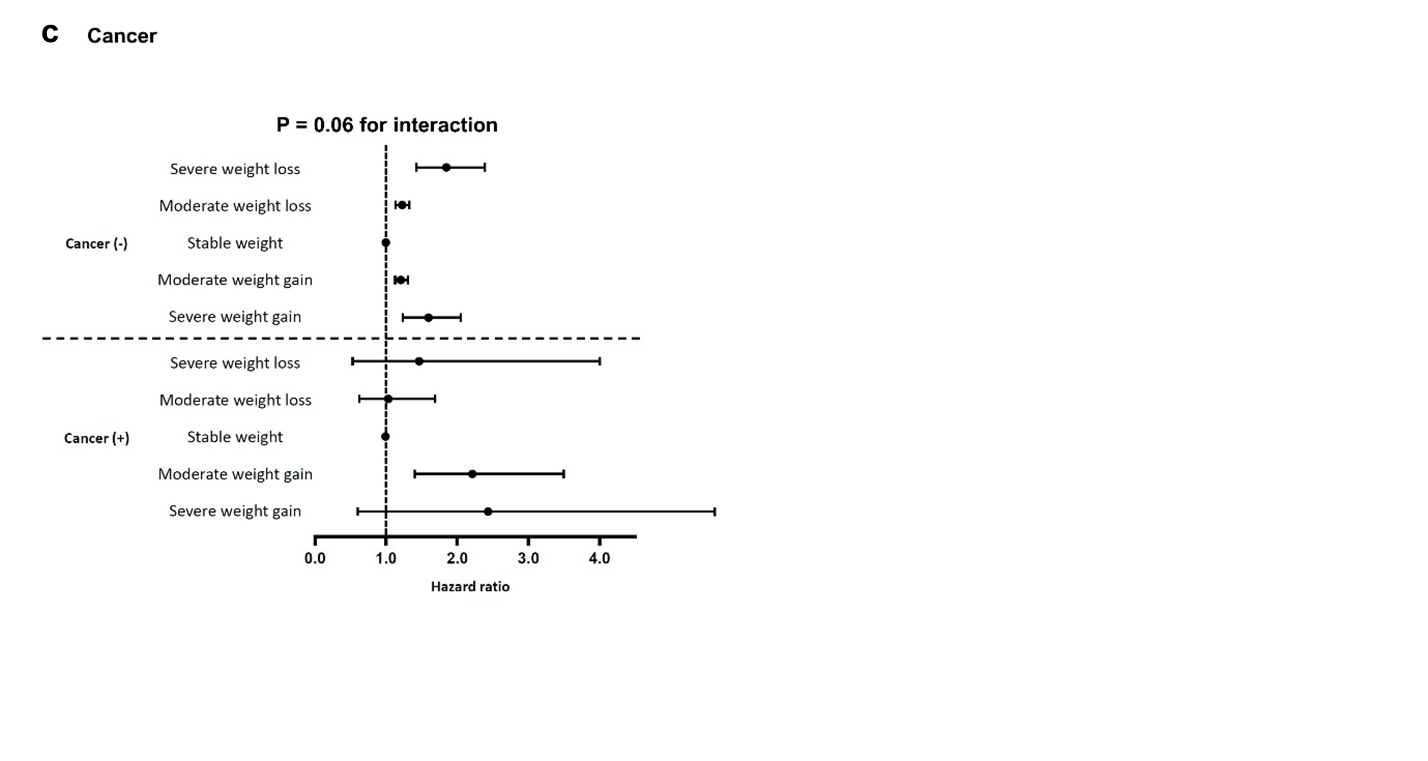


**Figure S4. Subgroup analyses according to BMI category.** Forrest plots of hazard ratios (HRs) and 95% confidence intervals (CIs) adjusted for age, sex, income (Q1), smoking, alcohol drinking, regular exercise, diabetes, hypertension, dyslipidemia, chronic kidney disease, depression, and waist circumference.

**
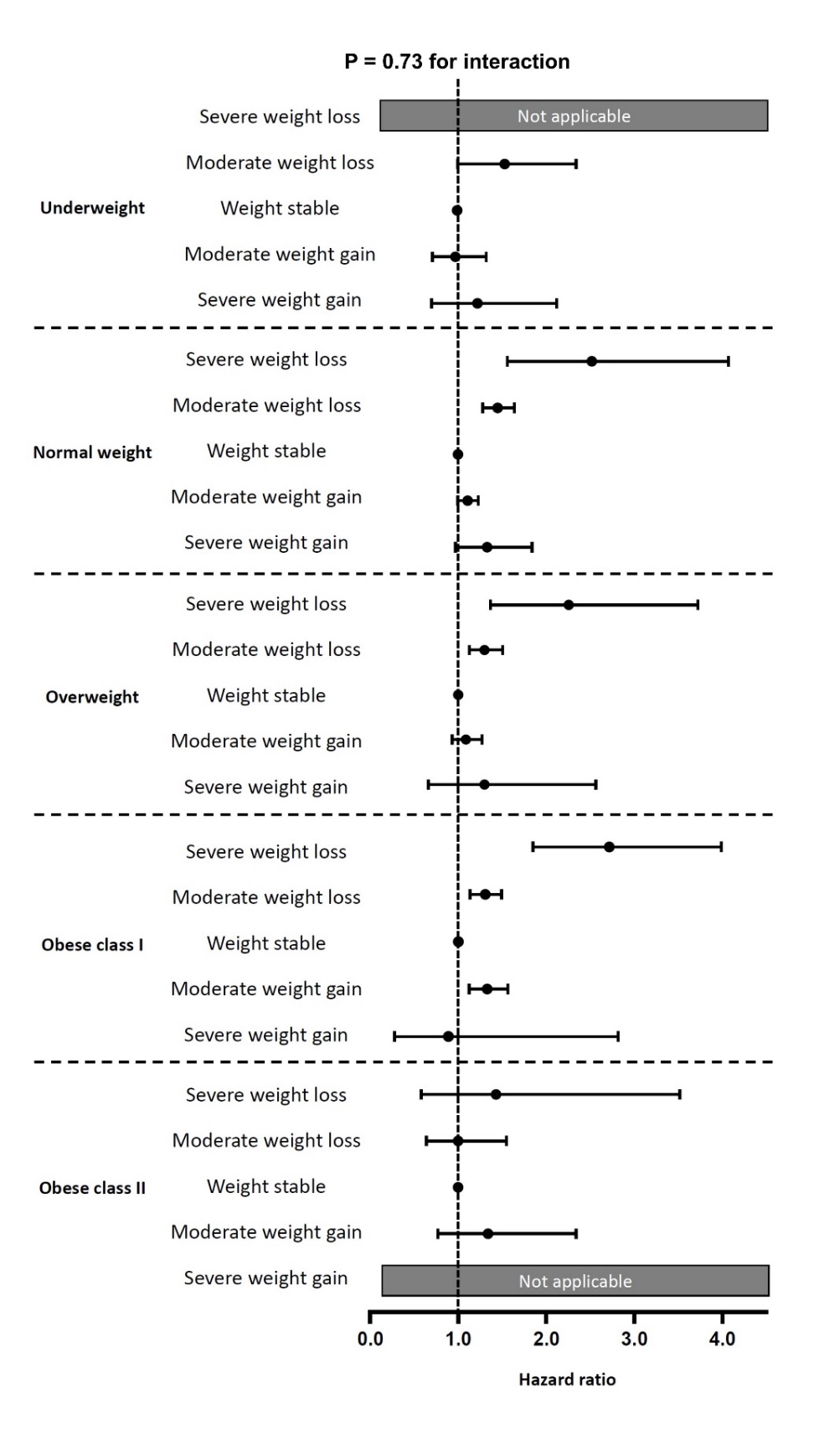
**

| **Table S1. Subgroup analysis for suicide mortality.** | | | | | | | |
| --- | --- | --- | --- | --- | --- | --- | --- |
|  |  | Weight change | Number | Event | IR  /1,000 PY | HR (95% CI)* | P for interaction |
| **Age** | **20–39 years** | <-15.0% | 3,349 | 7 | 0.19 | 1.35 (0.64–2.84) | 0.64 |
|  |  | -15.0 to <-5.0% | 60,294 | 137 | 0.20 | 1.25 (1.04–1.49) |  |
|  |  | -5.0 to <5.0% | 446,106 | 864 | 0.17 | 1 (Ref.) |  |
|  |  | 5.0 to <15.0% | 121,446 | 245 | 0.18 | 1.16 (1.00–1.33) |  |
|  |  | ≥15.0% | 9,724 | 18 | 0.16 | 1.45 (0.91–2.32) |  |
|  | **40–64 years** | <-15.0% | 4,306 | 28 | 0.60 | 2.19 (1.51–3.18) |  |
|  |  | -15.0 to <-5.0% | 116,316 | 406 | 0.31 | 1.27 (1.14–1.41) |  |
|  |  | -5.0 to <5.0% | 914,676 | 2,344 | 0.23 | 1 (Ref.) |  |
|  |  | 5.0 to <15.0% | 136,355 | 402 | 0.26 | 1.26 (1.14–1.40) |  |
|  |  | ≥15.0% | 6,461 | 28 | 0.39 | 1.88 (1.30–2.73) |  |
|  | **≥65 years** | <-15.0% | 3,058 | 29 | 1.13 | 1.63 (1.13–2.36) |  |
|  |  | -15.0 to <-5.0% | 43,130 | 289 | 0.69 | 1.13 (0.99–1.29) |  |
|  |  | -5.0 to <5.0% | 204,897 | 1,168 | 0.55 | 1 (Ref.) |  |
|  |  | 5.0 to <15.0% | 30,574 | 196 | 0.67 | 1.17 (1.00–1.36) |  |
|  |  | ≥15.0% | 2,833 | 18 | 0.69 | 1.29 (0.81–2.06) |  |
| **Sex** | **Male** | <-15.0% | 4,632 | 37 | 0.80 | 1.54 (1.12–2.14) | 0.23 |
|  |  | -15.0 to <-5.0% | 114,786 | 636 | 0.52 | 1.26 (1.15–1.37) |  |
|  |  | -5.0 to <5.0% | 946,221 | 3,503 | 0.34 | 1 (Ref.) |  |
|  |  | 5.0 to <15.0% | 168,786 | 664 | 0.36 | 1.24 (1.14–1.35) |  |
|  |  | ≥15.0% | 9,339 | 50 | 0.50 | 1.72 (1.30–2.27) |  |
|  | **Female** | <-15.0% | 6,081 | 27 | 0.42 | 2.49 (1.70–3.66) |  |
|  |  | -15.0 to <-5.0% | 104,954 | 196 | 0.17 | 1.14 (0.98–1.34) |  |
|  |  | -5.0 to <5.0% | 619,458 | 873 | 0.13 | 1 (Ref.) |  |
|  |  | 5.0 to <15.0% | 119,589 | 179 | 0.13 | 1.19 (1.02–1.40) |  |
|  |  | ≥15.0% | 9,679 | 14 | 0.13 | 1.33 (0.78–2.25) |  |
| **Diabetes** | **No** | <-15.0% | 9,212 | 53 | 0.55 | 1.92 (1.46–2.52) | 0.03 |
|  |  | -15.0 to <-5.0% | 194,428 | 718 | 0.34 | 1.28 (1.18–1.39) |  |
|  |  | -5.0 to <5.0% | 1,432,793 | 3,773 | 0.27 | 1 (Ref.) |  |
|  |  | 5.0 to <15.0% | 269,047 | 764 | 0.26 | 1.27 (1.17–1.37) |  |
|  |  | ≥15.0% | 17,429 | 53 | 0.28 | 1.57 (1.20–2.06) |  |
|  | **Yes** | <-15.0% | 1,501 | 11 | 0.79 | 1.51 (0.83–2.75) |  |
|  |  | -15.0 to <-5.0% | 25,312 | 114 | 0.44 | 0.95 (0.78–1.17) |  |
|  |  | -5.0 to <5.0% | 132,886 | 603 | 0.42 | 1 (Ref.) |  |
|  |  | 5.0 to <15.0% | 19,328 | 79 | 0.39 | 0.97 (0.77–1.23) |  |
|  |  | ≥15.0% | 1,589 | 11 | 0.70 | 1.87 (1.03–3.39) |  |
| **Depression** | **No** | <-15.0% | 10,111 | 55 | 0.52 | 1.86 (1.42–2.43) | 0.34 |
|  |  | -15.0 to <-5.0% | 210,267 | 720 | 0.31 | 1.20 (1.11–1.30) |  |
|  |  | -5.0 to <5.0% | 1,522,495 | 4,046 | 0.24 | 1 (Ref.) |  |
|  |  | 5.0 to <15.0% | 279,401 | 757 | 0.24 | 1.22 (1.12–1.31) |  |
|  |  | ≥15.0% | 18,273 | 53 | 0.27 | 1.53 (1.16–2.00) |  |
|  | **Yes** | <-15.0% | 602 | 9 | 1.65 | 1.83 (0.94–3.54) |  |
|  |  | -15.0 to <-5.0% | 9,473 | 112 | 1.13 | 1.46 (1.18–1.81) |  |
|  |  | -5.0 to <5.0% | 43,184 | 330 | 0.71 | 1 (Ref.) |  |
|  |  | 5.0 to <15.0% | 8,974 | 86 | 0.89 | 1.40 (1.10–1.78) |  |
|  |  | ≥15.0% | 745 | 11 | 1.45 | 2.31 (1.27–4.21) |  |
| **Cancer** | **No** | <-15.0% | 10,183 | 60 | 0.57 | 1.85 (1.43–2.39) | 0.06 |
|  |  | -15.0 to <-5.0% | 215,443 | 812 | 0.35 | 1.23 (1.14–1.33) |  |
|  |  | -5.0 to <5.0% | 1,547,312 | 4,305 | 0.25 | 1 (Ref.) |  |
|  |  | 5.0 to <15.0% | 285,222 | 818 | 0.26 | 1.21 (1.13–1.31) |  |
|  |  | ≥15.0% | 18,781 | 62 | 0.30 | 1.60 (1.24–2.05) |  |
|  | **Yes** | <-15.0% | 530 | 4 | 0.83 | 1.47 (0.54–4.01) |  |
|  |  | -15.0 to <-5.0% | 4,297 | 20 | 0.48 | 1.04 (0.63–1.70) |  |
|  |  | -5.0 to <5.0% | 18,367 | 71 | 0.37 | 1 (Ref.) |  |
|  |  | 5.0 to <15.0% | 3,153 | 25 | 0.77 | 2.22 (1.41–3.50) |  |
|  |  | ≥15.0% | 237 | 2 | 0.92 | 2.44 (0.61–9.86) |  |
| **BMI in 2007** | **<18.5 kg/m^2^** | <-15.0% | 65 | 0 | 0.00 | Not applicable | 0.73 |
|  |  | -15.0 to <-5.0% | 3,757 | 25 | 0.66 | 1.54 (1.01–2.35) |  |
|  |  | -5.0 to <5.0% | 48,793 | 161 | 0.30 | 1 (Ref.) |  |
|  |  | 5.0 to <15.0% | 16,469 | 55 | 0.30 | 0.98 (0.72–1.33) |  |
|  |  | ≥15.0% | 2,762 | 14 | 0.48 | 1.23 (0.71–2.13) |  |
|  | **18.5 to ≤ 22.9 kg/m^2^** | <-15.0% | 2,101 | 17 | 0.85 | 2.52 (1.56–4.07) |  |
|  |  | -15.0 to <-5.0% | 71,409 | 312 | 0.40 | 1.45 (1.28–1.64) |  |
|  |  | -5.0 to <5.0% | 593,683 | 1,699 | 0.26 | 1 (Ref.) |  |
|  |  | 5.0 to <15.0% | 144,182 | 431 | 0.27 | 1.11 (1.00–1.23) |  |
|  |  | ≥15.0% | 11,328 | 39 | 0.32 | 1.33 (0.97–1.84) |  |
|  | **23.0 to ≤**  **24.9 kg/m^2^** | <-15.0% | 2,373 | 15 | 0.62 | 2.30 (1.38–3.82) |  |
|  |  | -15.0 to <-5.0% | 56,004 | 210 | 0.34 | 1.31 (1.13–1.52) |  |
|  |  | -5.0 to <5.0% | 407,519 | 1,189 | 0.26 | 1 (Ref.) |  |
|  |  | 5.0 to <15.0% | 65,765 | 179 | 0.24 | 1.09 (0.93–1.28) |  |
|  |  | ≥15.0% | 2,754 | 8 | 0.26 | 1.31 (0.65–2.62) |  |
|  | **25.0 to ≤ 29.9 kg/m^2^** | <-15.0% | 4,542 | 27 | 0.57 | 2.68 (1.83–3.93) |  |
|  |  | -15.0 to <-5.0% | 77,876 | 261 | 0.31 | 1.30 (1.13–1.48) |  |
|  |  | -5.0 to <5.0% | 470,299 | 1,221 | 0.23 | 1 (Ref.) |  |
|  |  | 5.0 to <15.0% | 56,444 | 164 | 0.26 | 1.32 (1.12–1.55) |  |
|  |  | ≥15.0% | 1,963 | 3 | 0.14 | 0.89 (0.29–2.78) |  |
|  | **≥30.0 kg/m^2^** | <-15.0% | 1,632 | 5 | 0.28 | 1.43 (0.58–3.52) |  |
|  |  | -15.0 to <-5.0% | 10,694 | 24 | 0.20 | 1.00 (0.64–1.55) |  |
|  |  | -5.0 to <5.0% | 45,385 | 106 | 0.21 | 1 (Ref.) |  |
|  |  | 5.0 to <15.0% | 5,515 | 14 | 0.23 | 1.34 (0.77–2.34) |  |
|  |  | ≥15.0% | 211 | 0 | 0.00 | Not applicable |  |
| *Adjusted for age, sex, income (Q1), smoking, alcohol drinking, regular exercise, diabetes, hypertension, dyslipidemia, chronic kidney disease, depression, and weight at the 2009 screening (Except for the BMI subgroup analysis, in which waist circumference was used instead of weight at the 2009 screening). BMI denotes body mass index, CI confidence interval, HR hazard ratio, IR incidence rate, and PY person-years. | | | | | | | |
